# Genomic Signatures of Domestication in European Seabass (*Dicentrarchus labrax* L.) Reveal a Potential Role for Epigenetic Regulation in Adaptation to Captivity

**DOI:** 10.1002/ece3.70512

**Published:** 2024-12-03

**Authors:** Aristotelis Moulistanos, Konstantinos Papasakellariou, Ioannis Kavakiotis, Konstantinos Gkagkavouzis, Nikoleta Karaiskou, Efthimia Antonopoulou, Alexandros Triantafyllidis, Spiros Papakostas

**Affiliations:** ^1^ Department of Genetics, Development & Molecular Biology, School of Biology, Faculty of Sciences Aristotle University of Thessaloniki Thessaloniki Greece; ^2^ Genomics and Epigenomics Translational Research (GENeTres), Center for Interdisciplinary Research and Innovation (CIRI‐AUTH) Balkan Center Thessaloniki Greece; ^3^ Department of Science and Technology International Hellenic University Thessaloniki Greece; ^4^ Department of Zoology, School of Biology, Faculty of Sciences Aristotle University of Thessaloniki Thessaloniki Greece

**Keywords:** artificial selection, domestication genetics, genome‐wide variation, interactome‐assisted pathway analysis, marine teleost

## Abstract

Genome scans provide a comprehensive method to explore genome‐wide variation associated with traits under study. However, linking individual genes to broader functional groupings and pathways is often challenging, yet crucial for understanding the evolutionary mechanisms underlying these traits. This task is particularly relevant for multi‐trait processes such as domestication, which are influenced by complex interactions between numerous genetic and non‐genetic factors, including epigenetic regulation. As various traits within the broader spectrum of domestication are selected in concert over time, this process offers an opportunity to identify broader functional overlaps and understand the integrated genetic architecture underlying these traits. In this study, we analyzed approximately 600,000 SNPs from a Pool‐Seq experiment comparing eight natural‐origin and 12 farmed populations of European seabass in the Mediterranean Sea region. We implemented two genome scan approaches and focused on genomic regions supported by both methods, resulting in the identification of 96 candidate genes, including nine CpG islands, which highligt potential epigenetic influences. Many of these genes and CpG islands are in linkage groups previously associated with domestication‐related traits. The most significantly overrepresented molecular function was “oxidoreductase activity”. Furthermore, a dense network of interactions was identified, connecting 22 of the candidate genes. Within this network, the most significantly enriched pathways and central genes were involved in “chromatin organization”, highlighting another potential epigenetic mechanism. Altogether, our findings underscore the utility of interactome‐assisted pathway analysis in elucidating the genomic architecture of polygenic traits and suggest that epigenetic regulation may play a crucial role in the domestication of European seabass.

## Graphical abstract

Our study identified 96 candidate genes and nine CpG islands potentially linked to domestication in European seabass, many of which are associated with traits influenced by both genetic and epigenetic factors. The most enriched molecular functions were “oxidoreductase activity” and “chromatin organization”, indicating that epigenetic regulation may play a critical role in shaping domestication processes. Overall, our findings demonstrate the value of combining genome scans with interactome‐assisted pathway analysis to uncover the complex genetic architecture underlying polygenic traits like domestication.

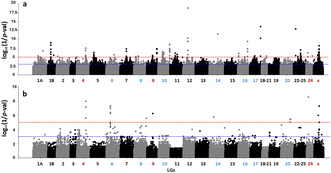

## 1 Introduction

Whole‐genome scans using high‐density single nucleotide polymorphisms (SNPs) have proven instrumental in uncovering patterns of genetic differentiation between populations, identifying candidate loci under selection, and elucidating the mechanisms driving local adaptation in natural populations and domestication in various species (Bigham et al. 2010; Cagan and Blass 2016; Chávez‐Galarza et al. 2013; Collevatti et al. 2019; Gkagkavouzis et al. 2021; Ma et al. 2015; Stölting et al. 2013; Tsartsianidou et al. 2021; Utsunomiya et al. 2013). This research has significantly advanced our understanding of the genetic basis of key life history and performance traits across diverse species, including reproductive strategies and lifespan, growth rate and development, survival, fecundity, parasite resistance, and mortality (Lai et al. 2016; McClure et al. 2010; Nkrumah et al. 2007; Nosrati et al. 2019; Tennessen et al. 2015; Valenzano et al. 2015; Van Kruistum et al. 2020; Wang et al. 2022; Yu et al. 2019; Zhou et al. 2022). Nevertheless, the characterization of genetic architecture for complex traits remains relatively sparse, focusing either on the identification of large‐effect loci or concluding that a highly polygenic architecture exists when no significant associations are detected (Uffelmann et al. 2021). To this end, among various methods, network‐based analysis is prominent for integrating genome scan results with functional interactions to pinpoint gene modules with association signals (Fagny and Austerlitz 2021; Jia and Zhao 2014; Liu et al. 2017; Tsare, Klapa, and Moschonas 2024). This is especially pertinent for multi‐trait processes like animal domestication, where unraveling the interconnected genetic mechanisms governing diverse phenotypes necessitates considering the complex interplay among multiple genes (e.g., Mabry et al. 2021). This knowledge is crucial for optimizing breeding and management strategies, and for appreciating adaptive responses to the challenges posed by climate change (Bernatchez et al. 2024; Fernandez‐Fournier, Lewthwaite, and Mooers 2021; Harrisson et al. 2014; Kelly 2019; Mariac et al. 2011).

Domestication is often viewed as an anthropogenic evolutionary experiment aimed at enhancing specific traits of interest, with growth and related traits frequently selected in commercial species. This process leads to rapid and profound genetic and phenotypic changes in the original founding populations, resulting in the emergence of domesticated genetic lineages characterized by improved growth and other desirable attributes (Ahmad et al. 2020; Milla et al. 2021; Purugganan 2019). Throughout human history, many animal and plant taxa have undergone domestication, several of which date back to ancient times (Ahmad et al. 2020; Purugganan 2019). Much more recently, fish species have also been domesticated by humans (Teletchea 2015). Due to their recent domestication history, farmed fish often show limited differentiation compared to natural‐origin individuals or populations across various species (López, Neira, and Yáñez 2014; Gkagkavouzis et al. 2021). This pattern represents a unique challenge for studying the genetic basis of fish domestication, potentially involving higher levels of genetic diversity or less well‐defined genetic architectures due to their shorter periods of artificial selection (Lorenzen, Beveridge, and Mangel 2012; Teletchea 2021). Nonetheless, studies have documented remarkable responses in life‐history traits regarding domestication in fish, often observed within just a few or even a single generation. This underscores the dynamic and rapidly evolving processes involved in fish domestication (Howe et al. 2024; Milla et al. 2021; Nguyen 2016). Traits such as growth, immune response, and reproduction are frequently targeted through artificial selection in farmed fish, from initial captive farming to specialized breeding programs (Milla et al. 2021; Teletchea 2021). Growth, often the primary focus, is evaluated using measurable parameters like body length and weight, which demonstrate moderate to high heritability (Chavanne et al. 2016; Gong et al. 2022; Yue 2014). Overall, selective breeding programs in fish have resulted in an impressive 12.5% average genetic gain per generation, specifically regarding growth improvement (Gjedrem, Robinson, and Rye 2012).

Despite advances in DNA sequencing technology, the genomic architecture of traits targeted by domestication remains elusive in several instances, even among commercially significant fish species with varying diets and temperature preferences (Ao et al. 2015; Boglione et al. 2013; D'Ambrosio et al. 2020; Mobley et al. 2021). For instance, Atlantic salmon (*Salmo solar* Linnaeus 1758) and rainbow trout (*Oncorhynchus mykiss* Walbaum 1792) are cold‐water, piscivorous species, while large yellow croaker (*Larimichthys crocea* Richardson 1846) and European seabass (*Dicentrarchus labrax* Linnaeus 1758), are warm‐water, piscivorous species. In contrast, the gilthead seabream (*Sparus aurata* Linnaeus 1758) is a warm‐water species with omnivorous feeding habits. The challenges in identifying genetic loci linked to such life‐history traits stem from their polygenic nature, but also from the significant interactions between genetic and non‐genetic factors, including epigenetic modifications (Koch, Nuetzel, and Narum 2023; Mobley et al. 2021; Mohamed et al. 2019; Sinclair‐Waters et al. 2020). Only a handful of cases have shown that a single genetic locus can significantly impact phenotypic variation in teleost fish. Notably, the *vgll3* gene accounts for 39% of the variation in age at maturity in Atlantic salmon (*Salmo salar* L. 1758; Barson et al. 2015). The *greb1l* gene influences migration patterns, explaining spring or fall migration phenotypes in Chinook salmon (*Oncorhynchus tshawytscha* Walbaum 1792) populations (Thompson et al. 2019) and 50% of the migration variance in coastal Steelhead (*Oncorhynchus mykiss* Walbaum 1792) populations (Willis et al. 2020). Similarly, the *mc4r* gene affects size variation in male *Xiphophorus* fishes; its functional copy numbers on chromosome Y delay puberty and promote larger body size, favored by females (Lampert et al. 2010). In most other cases, traits targeted by domestication have been found to be polygenic, reflecting the complex interplay of multiple genetic factors that contribute to trait variation. This complexity makes it challenging for genome scans to pinpoint specific genes or functions (Lagarde et al. 2023; Rey et al. 2020; Sinclair‐Waters et al. 2020; Whiting et al. 2022). Intriguingly, studies primarily using bisulfite sequencing have also reported substantial epigenetic differences between farmed and natural‐origin fish (Koch, Nuetzel, and Narum 2023). These differences include variations in methylation patterns across various genomic regions, which have been associated with diverse biological functions such as immune response, metabolism, and development. For example, hatchery‐origin fish exhibit greater hypermethylation than their natural‐origin counterparts, with specific regions linked to critical functions like ion homeostasis, neuromuscular regulation, and stress response (Le Luyer et al. 2017; Leitwein et al. 2021, 2022; Nilsson et al. 2021). It thus seems that the interplay between genetic and epigenetic factors is more complex than previously thought, influencing a broad range of traits in domesticated fish.

The European seabass is a highly economically important fish species in Europe, justifying the focus on several selective breeding programs (Teletchea 2021). Initial trials of farming European seabass in captivity began around the 1970s, with the first selective breeding programs implemented by the 1990s (Janssen et al. 2017). The domestication process of European seabass is thus relatively recent (Vandeputte, Gagnaire, and Allal 2019), and a significant reduction in the effective population size of farmed populations was reported only eight to nine generations ago (Saura et al. 2021). Population structure analysis in the Mediterranean Sea has also revealed clear differentiation between farmed and natural‐origin populations of European seabass suggesting significant genetic divergence due to farming practices (Villanueva et al. 2022). As with all commercially important fish species, selective breeding programs for European seabass have primarily targeted growth performance (Janssen et al. 2017). Previous studies using quantitative trait locus (QTL) analysis have identified genomic regions associated with various domestication‐related traits such as growth performance, stress tolerance, and disease resistance (Chatziplis et al. 2020; Louro et al. 2016; Massault et al. 2010). However, the specific genes and biological functions influenced by the domestication process in European seabass have not been extensively documented to date. Since the whole‐genome sequencing and annotation of the European seabass became available (Tine et al. 2014), it is possible to conduct population genomics research in this direction.

In this study, we aimed to investigate the genome‐wide signatures of domestication in European seabass. We analyzed Illumina Pool‐Seq data from 12 farmed and eight natural‐origin populations of European seabass across the Mediterranean region. The dataset was produced by Peñaloza et al. (2021), and was originally used to develop a SNP chip for population genomic analyses in gilthead seabream and European seabass. In a recent study, (Moulistanos et al. 2023), we leveraged this dataset to explore the impact of domestication on the genetic variation of two chromosomes in European seabass, which contain the candidate genes *six6* and *vgll3*, previously associated with maturation in Atlantic salmon (*Salmo salar*) (Barson et al. 2015; Sinclair‐Waters et al. 2020). Our findings revealed genomic regions with high‐level differentiation between farmed and natural‐origin populations in these two chromosomes, highlighting the potential of this dataset to identify targets of selection during domestication (Moulistanos et al. 2023). Expanding upon these insights, the current work extends the analysis to a genome‐wide scale, enabling a comprehensive exploration of genes and functions affected by the domestication process in European seabass. Additionally, we devised an approach to investigate the network of functional interactions to gain a comprehensive understanding of the genomic architecture of domestication. This approach aimed to encompass the intricate interactions between candidate loci with purported small effects, assessing whether these genes are tightly interconnected within functional pathways. By doing so, we sought to determine if, despite their individual small effects, these loci collectively contribute to an overrepresented functional pathway. Altogether, we provide a comprehensive overview of the genomic architecture of domestication in European seabass, while also presenting a method to explore genes and gene networks associated with polygenic traits relevant to domestication, such as growth, stress response, and behavior that extend beyond large‐effect genes.

## 2 Materials and Methods

**FIGURE 1:**
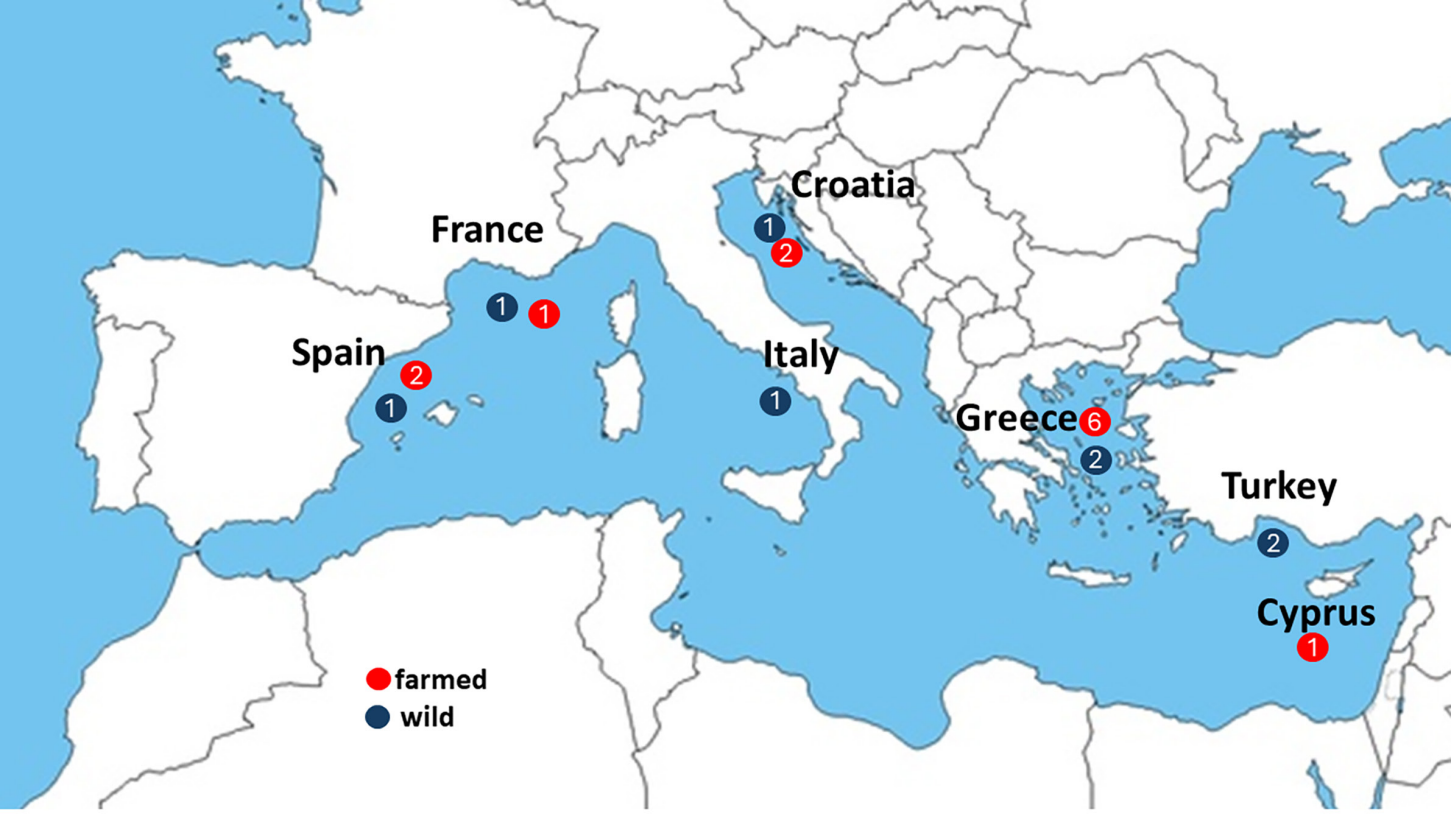
Geographical distribution of farmed and nature‐origin populations in the Mediterranean region.

We utilized whole‐genome sequencing data from pooled samples (Pool‐Seq) of 12 farmed and eight natural‐origin populations of European seabass. These populations originated from seven countries within the Mediterranean Sea region (Peñaloza et al. 2021; Table 1; Figure 1). We excluded four populations from the original dataset comprising 14 farmed and 10 natural‐origin populations based on previous population structure analyses conducted by Peñaloza et al. (2021) and Villanueva et al. (2022). Specifically, we excluded two farmed populations with unexpectedly high effective population size (N_e_ > 1.000), one natural‐origin population with a very low effective population size (N_e_ = 4.8), and one population showing evidence of genetic admixture with the Atlantic lineage of European seabass (Peñaloza et al. 2021; Villanueva et al. 2022). Farmed populations with N_e_ > 1000 could introduce noise in identifying domestication‐affected regions due to their genetic proximity to natural‐origin populations. Their high N_e_ could suggest that selection is still in its early stages or that broodstock is being renewed with natural‐origin fish, which is a common practice in Mediterranean aquaculture (Villanueva et al. 2022). Additionally, it is important to note that the N_e_ of most farmed European seabass populations have undergone a significant reduction due to domestication bottlenecks approximately 10 generations ago (Saura et al. 2021). The natural‐origin population with low N_e_ (N_e_ = 4.8) had a different genetic makeup likely influenced by farmed escapees, and its inclusion could lead to spurious signals of selection (Villanueva et al. 2022). Finally, the natural‐origin population with Atlantic admixture could skew differentiation analyses due to the introduction of alleles from distinct genetic backgrounds. The exclusion of populations with unusual effective population sizes (N_e_) and evidence of genetic admixture was essential to ensure the accuracy of our analyses and to improve the reliability of identifying genomic regions with significant differential allele frequencies. Any uncertainties in the genetic history of the populations studied could compromise our conclusions by potentially masking the domestication signal.

**TABLE 1:** Classification of Pool‐Seq European seabass samples and population identities as either farmed or natural‐origin in the studied Mediterranean countries (adapted from Peñaloza et al. 2021).

Status	Population identity^a^	Country of origin	Number of individuals per pool	Technical replicates
Farmed	fFRA_1	France	12	1
	fSPA_2	Spain	25	2
	fSPA_3	Spain	25	2
	fCRO_5	Croatia	25	2
	fCRO_6	Croatia	25	2
	fGRE_7	Greece	25	2
	fGRE_8	Greece	25	2
	fGRE_9	Greece	25	2
	fGRE_10	Greece	25	2
	fGRE_11	Greece	25	2
	fGRE_12	Greece	25	1
	fCYP_13	Cyprus	25	2
Natural‐origin	wFRA_1	France	25	2
	wSPA_2	Spain	11	1
	wITA_4	Italy	25	2
	wCRO_8	Croatia	12	1
	wGRE_6	Greece	25	2
	wGRE_7	Greece	25	2
	wTUR_9	Turkey	25	2
	wTUR_10	Turkey	25	2

*Note:* The effective population size (N_e_), as estimated by Villanueva et al. (2022) for the same populations, averaged 23.7 and ranged from 8.9 to 49.1 in the farmed populations. In contrast, the natural‐origin populations had a significantly larger Ne, averaging 468.8 and ranging from 37 to over 1000.

^a^ Labeling was done according to Peñaloza et al. (2021).

### 2.1 Read Mapping

We obtained the Pool‐Seq data for European seabass each population from the NCBI Sequence Read Archive under the accession ID PRJEB40423. To ensure data quality, we filtered the sequences using Trimmomatic (v. 0.38, Bolger, Lohse, and Usadel 2014) with the following parameters in paired‐end mode: ILLUMINACLIP: TruSeq3‐PE.fa:2:30:10; LEADING:5; TRAILING:5; SLIDINGWINDOW:3:15; MINLEN:100. Subsequently, we mapped the filtered reads to the reference assembly (GCA_000689215.1) using the bwa mem algorithm (Li and Durbin 2009). Finally, we extracted only properly paired reads with a mapping quality of at least 15 (equivalent to a maximum 3% misalignment probability) using samtools (v. 9.2.0, Li et al. 2009).

### 2.2 SNP Genotyping

To ensure accurate genotype frequencies, we processed the properly paired reads from each population in Table 1 by sorting and merging them between technical replicates using samtools. Subsequently, we used bam‐readcount v.1.0 (Khanna et al. 2022) to obtain read counts for each genomic position with mapped reads. We applied an AWK script to filter these positions, requiring a minimum read depth of 25 counts. This threshold was determined through computer simulations involving 1 million resampling events from a pool of 25 samples. The simulations demonstrated that a read depth of 25 counts adequately represented at least half of all possible genotypes within each population pool, with the lower 95% confidence limit ensuring that at least 13 samples were represented. Allele frequencies below 1% were excluded to mitigate potential sequencing errors and incorrect mappings, aligning with common practice in population genomic analyses (Linck and Battey 2019). Finally, we employed an in‐house Python function to identify biallelic SNPs and their corresponding genotypes. The Python scripts used for simulations and SNP typing are available at the GitHub link provided in the Data Availability section.

### 2.3 PCA & Genome Scan Analyses

To examine and characterize differentiation between the studied farmed and natural‐origin populations of European seabass, Principal Component Analysis (PCA) was performed using the Python package “sklearn.” Allele frequencies between farmed and natural‐origin populations were compared using two programs: PoPoolation2 (Kofler et al. 2011) and BayPass v. 2.1 (Gautier 2015), both of which accommodate Pool‐Seq experimental designs. In‐house Python code was utilized to produce input files for these programs.

PoPoolation2 was used to calculate pairwise *F*_ST_, representing the proportion of total genetic variance contained within subpopulations relative to the total genetic variance. We specifically focused on the *F*_ST_ differences between farmed and wild populations and calculated the average *F*_ST_ for each SNP. Statistical significance between farmed and natural‐origin populations for each SNP was determined using Fisher's exact test.

BayPass was executed in Pool‐Seq mode with a burn‐in of 10,000 iterations, which is double the default value. We recorded 10,000 samples with thinning, which is the number of iterations between two recorded samples, set to the default value of 25, resulting in a post‐burn‐in MCMC chain length of 250,000 iterations. Other parameters were kept at their default values. BayPass was employed to calculate the XtX differentiation statistic between farmed and natural‐origin populations of European seabass determining its significance for each SNP. It should be noted that the XtX statistic is similar to *F*_ST_, but is corrected for the scaled covariance of population allele frequencies, thus providing estimates that are less sensitive to outlier populations (Günther and Coop 2013).

The *p*‐values produced by both programs were adjusted for multiple testing using the Benjamini–Hochberg method (Benjamini and Hochberg 1995), as implemented in the “stats” package in Python. The SNPs with adjusted *p*‐values lower than 10^−5^ in both PoPoolation2 and BayPass will be henceforth referred to as “highly suggestive,” whereas those with adjusted *p*‐values below 10^−3^ in one program and 10^−5^ in the other are referred to as “suggestive.”

### 2.4 Functional Enrichment Analyses

For each suggestive and highly suggestive SNP, information on neighboring genes or regulatory regions, such as CpG islands within a 100‐kilobase pair (Kbp) region on both sides, was extracted (Barson et al. 2015; Star et al. 2016). This was accomplished using genome annotations (*.gff3 files) from BioMart (Filename: Dicentrarchus_labrax. seabass_V1.0.105.gff3). Sequences of identified genes were downloaded from Ensembl seabass_V1.0 (GenBank assembly ID: GCA_000689215.1) and were used to identify better‐annotated zebrafish (*Danio rerio* Hamilton 1822) orthologs via local BLASTx using zebrafish UniProtKB/Swiss‐Prot identifiers (https://www.uniprot.org/blast). In each case, the top BLASTx hit was selected, with a maximum *E*‐value threshold of 10^−3^. Two approaches were employed to describe the functional properties of the identified genes. Firstly, we conducted a classical GO enrichment analysis using the PANGEA tool (Hu et al. 2023) with Benjamini–Hochberg correction for multiple statistical tests, using the zebrafish as the reference genome (https://zfin.org/). This analysis aimed to detect GO terms in the categories of molecular function, biological process, and cellular component, enriched in our list of candidate genes. Secondly, we conducted a pathway enrichment analysis incorporating functional interaction data to enhance the robustness and depth of the pathway analysis (Fagny and Austerlitz 2021; Jia and Zhao 2014). We downloaded predicted functional couplings for zebrafish from the FunCoup v.5.0 database (Persson et al. 2021). We filtered these interactions to include only those with a predicted probability of 90% or higher. Previous research has shown that such functional couplings are strong predictors of true positive interactions, such as links between proteins of the same complex or proteins involved in the same metabolic pathway (Papakostas et al. 2014). We further filtered the interactions to include only those where our candidate genes were direct interactors. We identified networks through an iterative process of increasing degree connectivity of interacting partners, while assessing the significance of enrichment (via the Fisher exact test) in candidate genes. This enrichment within the network of direct interactions indicated a potential biological relatedness among the candidate genes. We also repeated this analysis for 1000 permutations, each time starting with a random set of genes of the same size as our candidate gene list drawn from the genome, to assess the degree to which the observed enrichment was greater than would be expected by chance. The resulted network was visualized using Cytoscape v. 3.10.2 (Shannon et al. 2003), and its topology analyzed with CentiScaPe (Scardoni et al. 2014). Our candidate genes involved in the network were used for pathway enrichment analysis using the PANGEA too in the zebrafish reference genome (Hu et al. 2023).

## 3 Results

**FIGURE 2:**
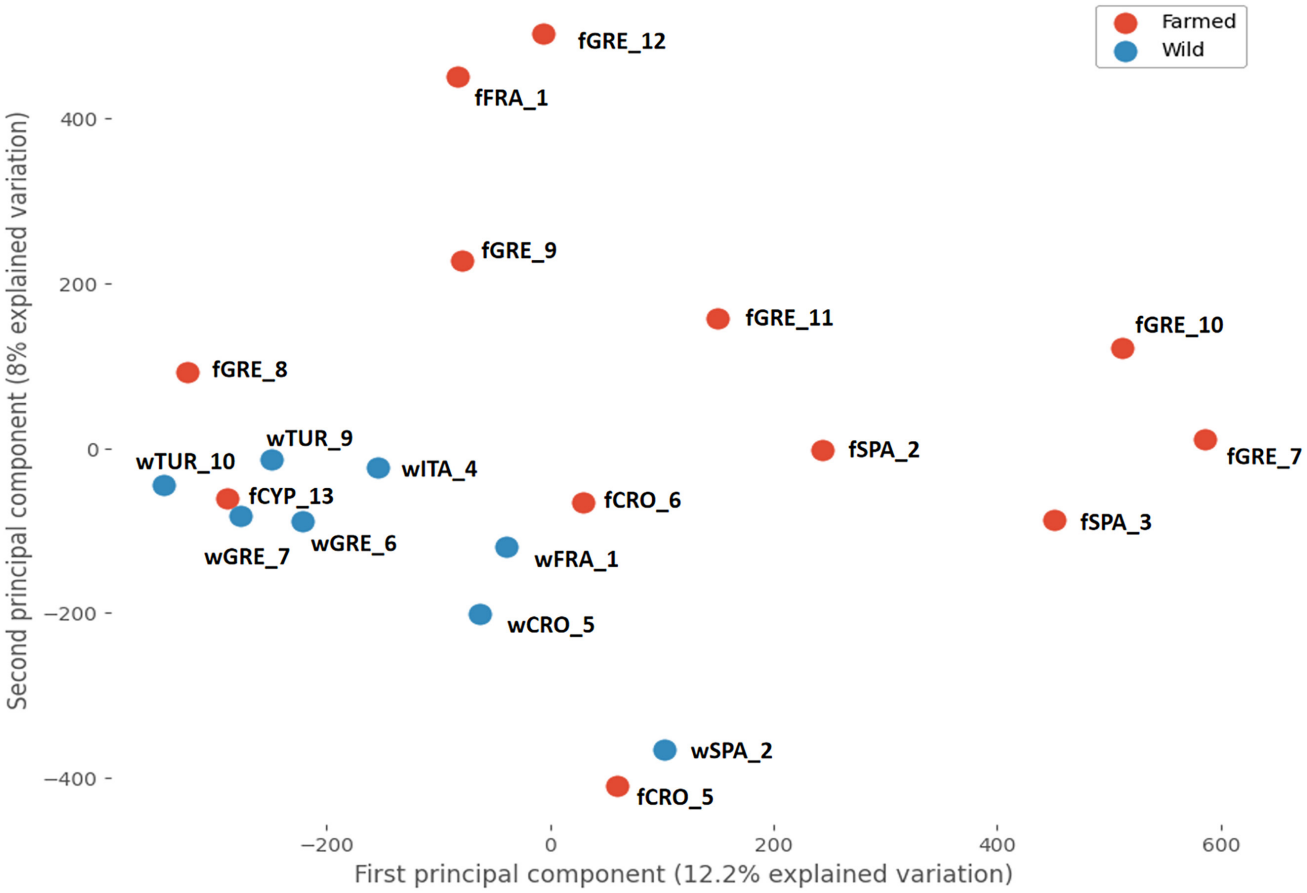
Principal component analysis (PCA) was conducted on 593,479 SNPs for the studied farmed and natural‐origin (wild) populations of European seabass across the Mediterranean region with information of each population ID based on Table 1.

We examined and analyzed the allele frequencies of 593,479 biallelic SNPs across the entire genome of European seabass. The PCA demonstrated good differentiation between farmed and natural‐origin populations (Figure 2). The first principal component accounted for 12.2% of the total variation, while the second principal component explained 8% of the variation. There was some overlap between farmed and natural‐origin populations, specifically involving four farmed populations (fGRE_8, fCRO_5, fCRO_6 and fCYP_13; Figure 2). Nevertheless, we included these populations in our analyses to ensure more conservative conclusions.

**FIGURE 3:**
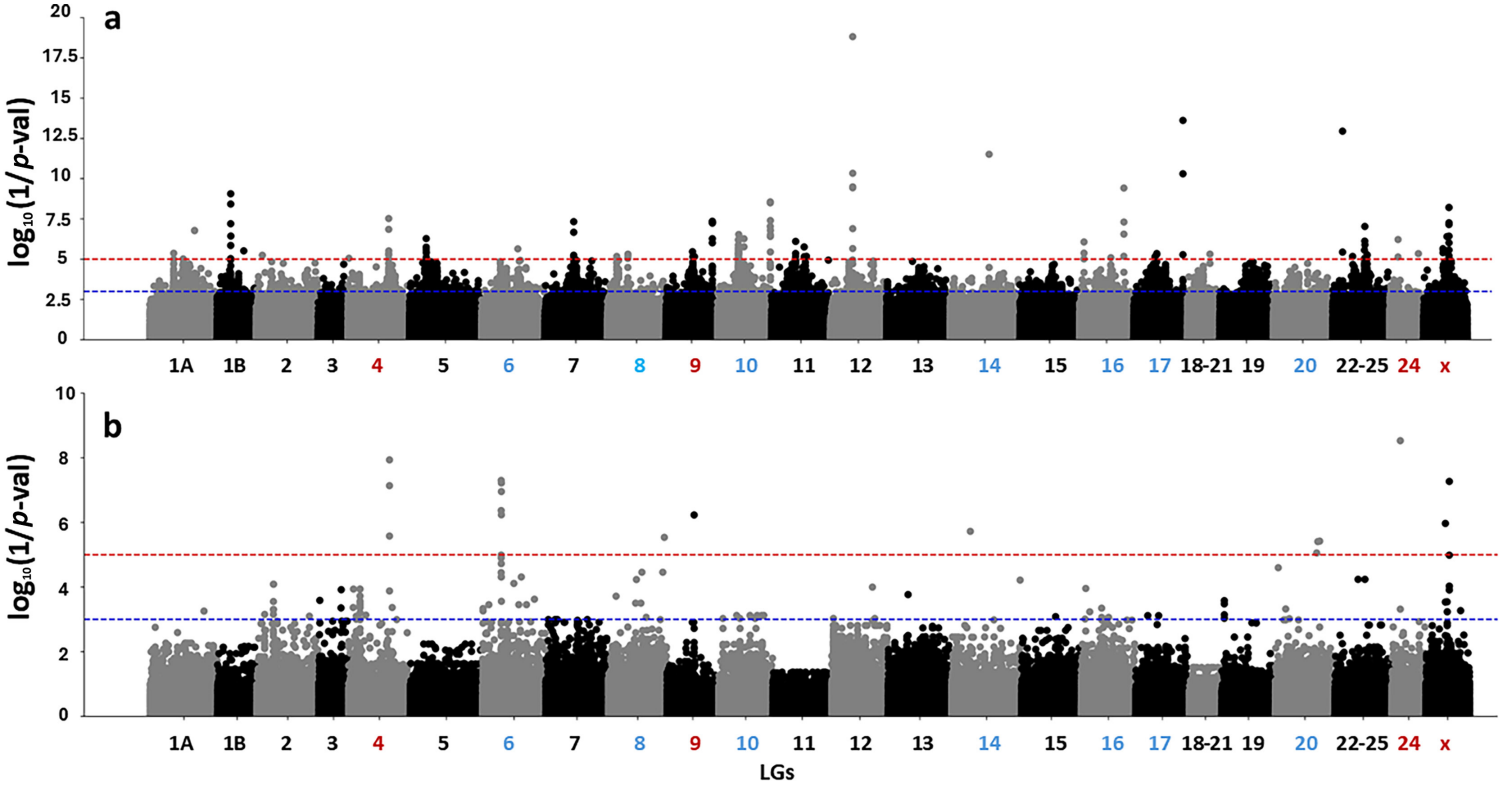
Manhattan plots depict the statistical significance of tests from the two genome scan methods across the European seabass genome. Panel “a” shows the log_10_(1/*p*‐val) of Fisher's exact test in *F*_ST_‐based method using PoPoolation2, and panel “b” displays the corresponding values from the Chi‐squared distribution in XtX‐based method using BayPass. *p*‐values were adjusted for multiple testing using the Benjamini–Hochberg method. Horizontal red and blue lines indicate thresholds for statistical significance at log_10_(1/*p*‐val), corresponding to *p* = 10^−5^ and *p* = 10^−3^, respectively. Linkage groups' (LG) names are labeled on the *x*‐axis, with red indicating LGs containing highly suggestive SNPs and with blue representing LGs with suggestive SNPs.

Both genome scan methods, Popoolation2 and BayPass, identified genomic regions with statistically significant differentiation between farmed and natural‐origin populations. We detected 17 differentiated SNPs across 11 linkage groups (LGs) categorized as either “suggestive” or “highly suggestive”, potentially involved in the domestication process (Figure 3). Among these, five SNPs were highly suggestive, located in four LGs (LG4, LG9, LG24, LGx), while the remaining 12 SNPs were suggestive, spanning across seven LGs (LG6, LG8, LG10, LG14, LG16, LG17, LG20). The *F*_ST_, XtX values, and their adjusted *p*‐values for each “suggestive” and “highly suggestive” SNP are detailed in Table S1.

Our review of annotations within a 100‐kilobase pair (kbp) window on both sides of each “suggestive” and “highly suggestive” peak identified 96 candidate genes and nine CpG islands potentially contributing to the domestication process in the studied species (Table S1). We identified six significant Gene Ontology (GO) terms significantly associated with these genes. The most multitudinous Molecular Function term from candidate genes was *oxidoreductase activity* (GO:0016491), with 12 out of 96 identified genes (*p*_adj_ = 5.025e−3) (Table 2). Additionally, three GO terms for Biological Processes, namely *exogenous drug catabolic process* (GO:0042738), *response to drug* (GO:0042493) and *xenobiotic metabolic process* (GO:0006805) showed the same adjusted *p*‐value (*p*_adj_ = 5.025e−3). These processes involved four of the candidate genes, namely *cyp2n13, cyp2p10, cyp2p6*, and *cyp2p7*, located on LG4.

**TABLE 2:** The three most significant Gene Ontology (GO) terms and molecular pathways associated with domestication process of European seabass.

Annotation	*p*_adj_	Identified genes	Associated LGs (gene number)
Oxidoreductase activity (GO:0016491)	5.025e−3	*aldh8a1; pyroxd1; cyp2p7; cyp2p6; hsd17b12a; tet3; txnrd2.2; cyp2n13; mmachc; alkbh3; hsd17b; cyp2p10*	LG4 (6); LG6 (2); LG20 (2); LG17 (1); LGx (1)
*Chromatin‐modifying enzymes* (R‐DRE‐3247509)	1.425e−4	*hist2h2l; h2ax1; smarcc1a; kat6a*	LG14 (2); LG16 (1); LG20 (1)
*Chromatin organization* (R‐DRE‐4839726)			

**FIGURE 4:**
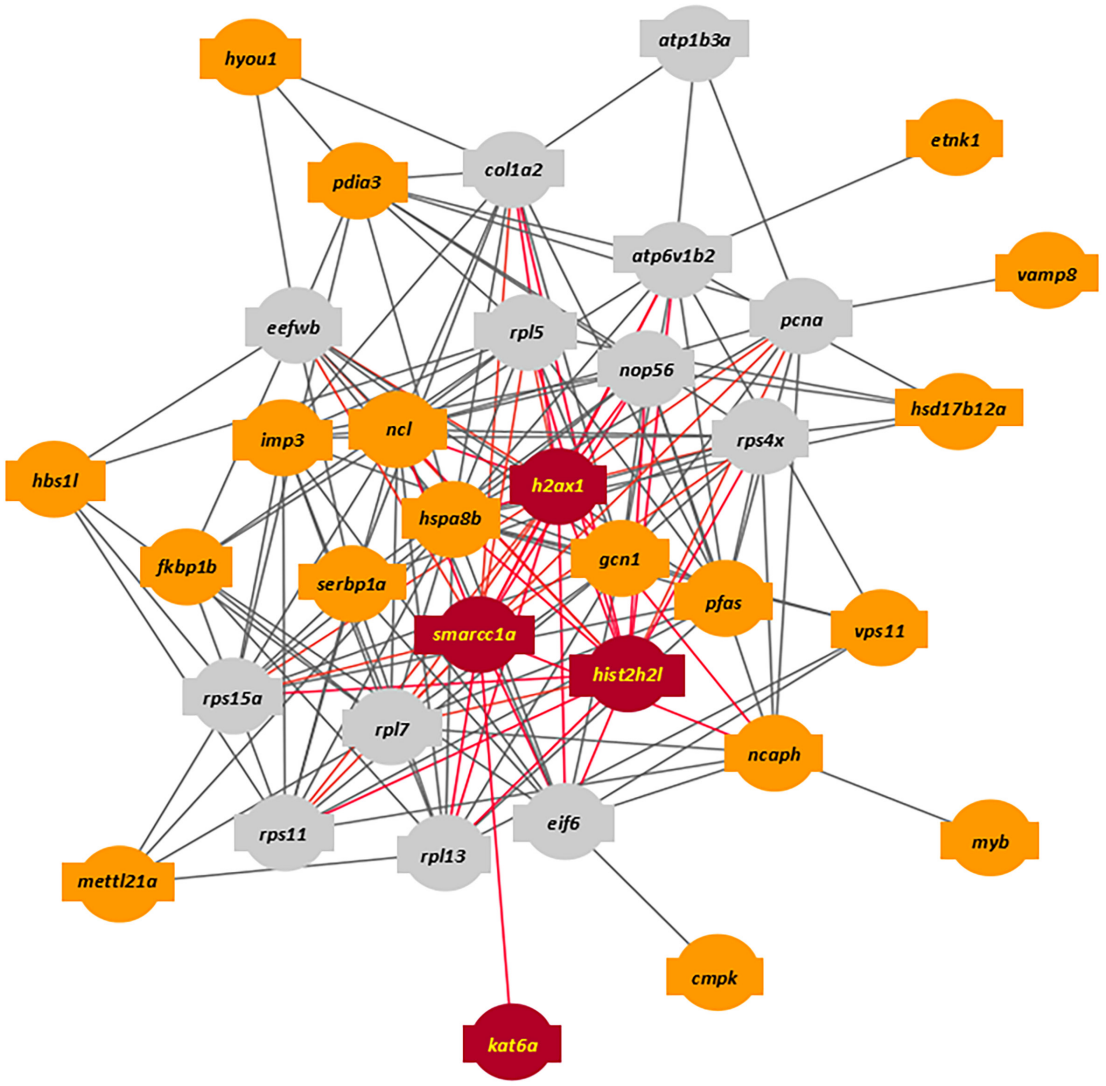
The highly interconnected network identified includes 22 candidate genes (denoted with orange and red color). Gray nodes represent 13 intermediate genes identified through functional couplings. Red nodes specifically highlight genes annotated with the significantly enriched pathways namely “Chromatin‐modifying enzymes” and “Chromatin organization.” *h2ax1*: H2A.X variant histone family member 1; *smarcc1a*: SWI/SNF related, matrix associated, Actin dependent regulator of chromatin, subfamily c, member 1a; *hist2h2l*: Histone 2, H2‐like; *kat6a*: Lysine acetyltransferase 6A.

**FIGURE 5:**
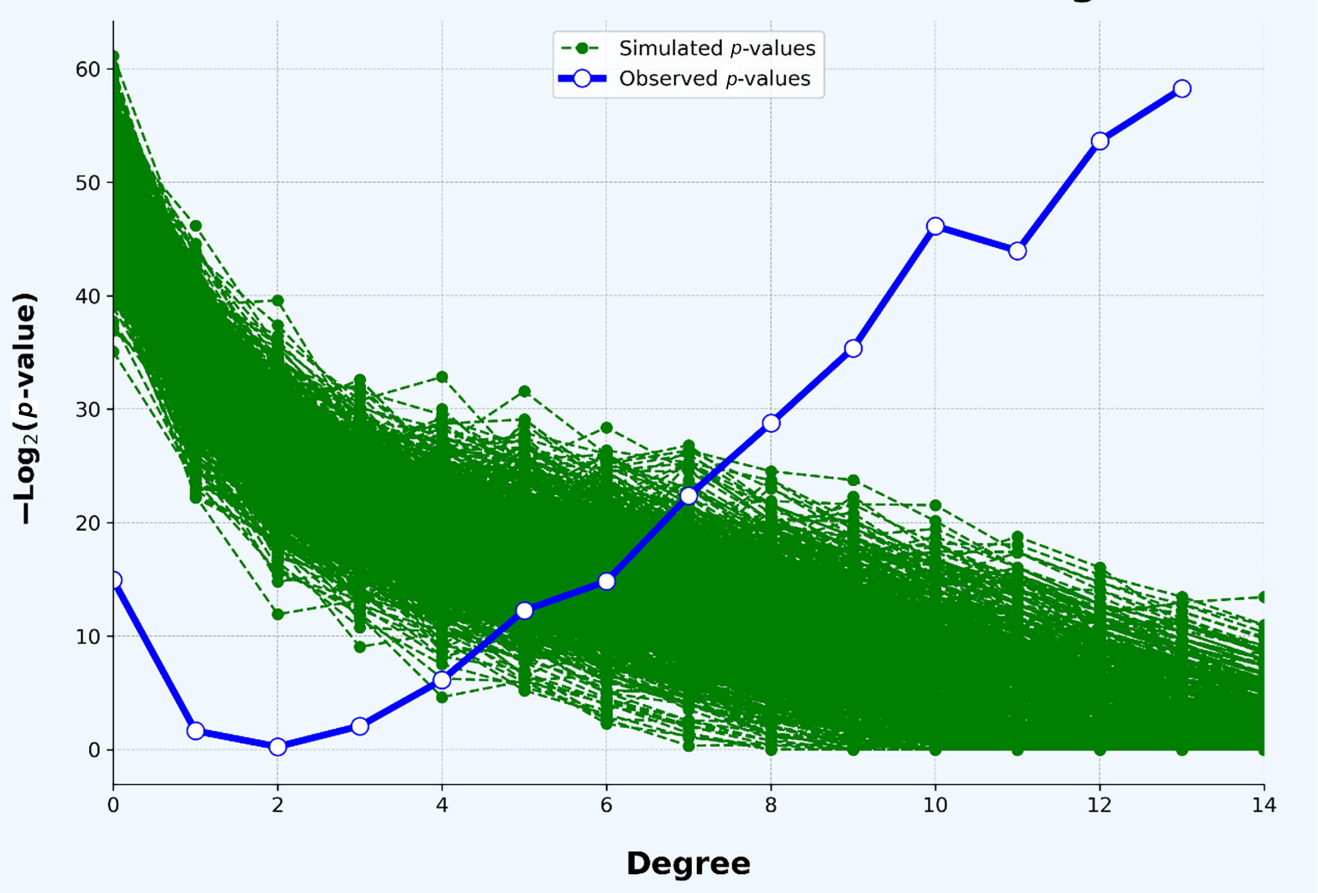
Comparison of observed and simulated enrichment in target genes based on their degree topological indices. The green lines represent the *p*‐values from 1000 simulations performed on random sets of genes of the same size drawn from the genome. The blue line indicates the observed *p*‐values of enrichment within our identified candidate genes.

**FIGURE 6:**
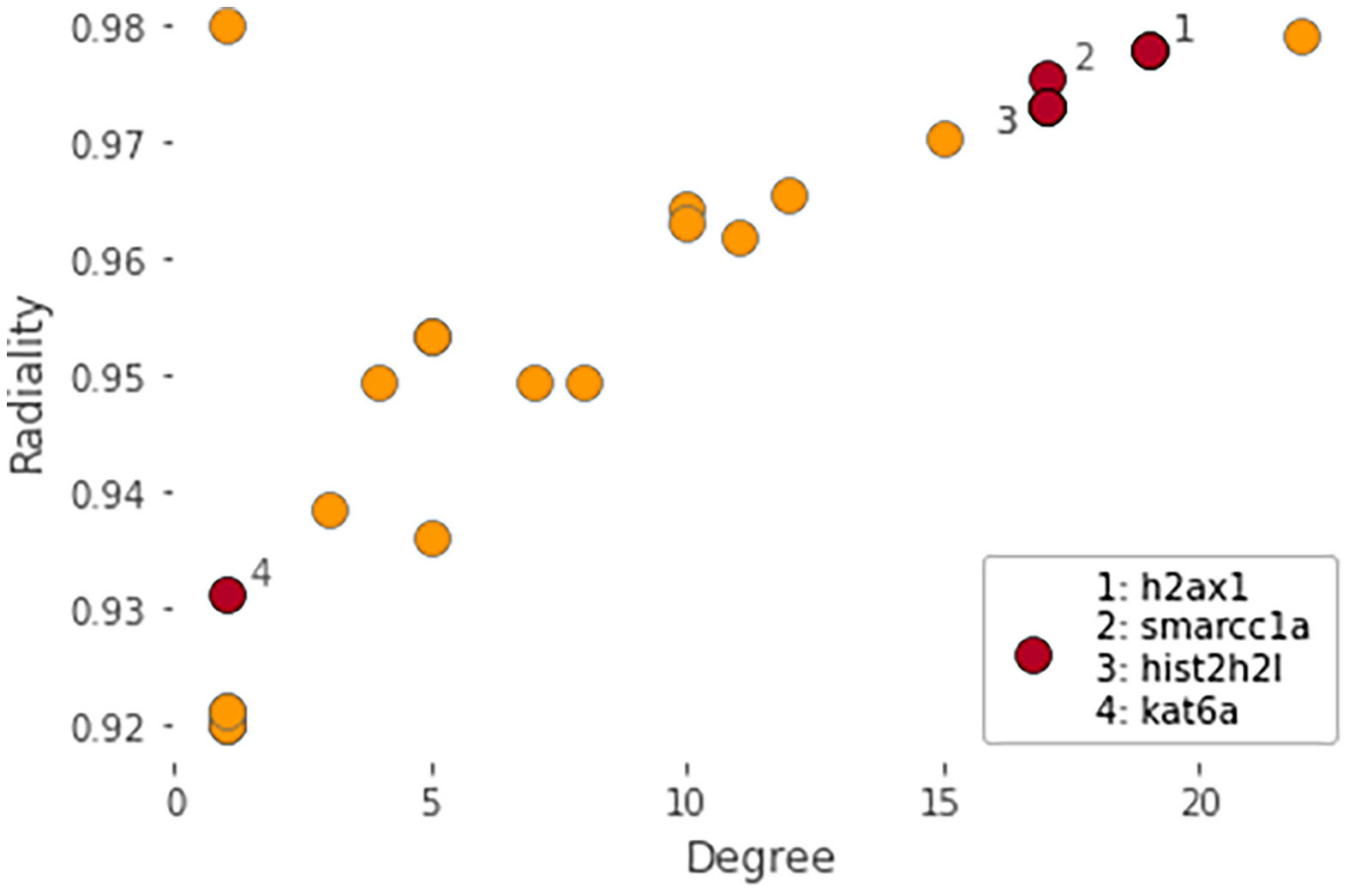
Scatter plot showing the distribution of the genes in the identified network based on their degree and radiality topological indices. The degree corresponds to the number of directly connected nodes; thus, a higher degree indicates a more central role in the network. Radiality, another node centrality index, was estimated by computing the shortest path between the given node and all other nodes in the network (higher centrality indicating a greater influence and potential within the network). Red points highlight the genes that participated in the most statistically significant enriched pathways namely “Chromatin‐modifying enzymes” and “Chromatin organization.” Gene names: *H2ax1*, H2A.X variant histone family member 1; *hist2h2l*, histone 2, H2‐like; *smarcc1a*, SWI/SNF related, matrix associated, Actin dependent regulator of chromatin, subfamily c, member 1a; *kat6a*, lysine acetyltransferase 6A.

A single tightly interconnected network of interactions was identified that included 22 of the 96 candidate genes and 13 interacting partners, each connected with 14 interactions (Figure 4). The enrichment of this network with candidate genes was estimated at 2.89e−18. The enrichment of the 1000 permutations on random sets of genes of the same size drawn from the genome rapidly decreased to non‐significance with increasing degree value (Figure 5), suggesting that our list of candidate genes does have biological connection. The two most significantly enriched pathways identified among the 22 candidate genes in the network were *chromatin‐modifying enzymes* (R‐DRE‐3247509; *p* = 1.425e−4) and *Chromatin organization* (R‐DRE‐4839726; *p* = 1.425e−4) (Table 2). Notably, genes associated with these pathways were among the top interconnected genes in the network, as indicated by the topological analysis (Figure 6).

## 4 Discussion

In this study, we thoroughly analyzed a comprehensive Pool‐Seq dataset of European seabass sampled across the Mediterranean region, and we have provided an overview of the functional properties of the multi‐trait polygenic architecture of domestication in this species. Using a high‐density set of nearly 600 K SNPs, we investigated the genomic landscape of differentiation between several farmed and natural‐origin populations of this commercially very important fish species. Through traditional gene set enrichment analysis, we interrogated the Gene Ontologies of the list of candidate genes associated with domestication. We further rigorously scrutinized predicted functional interactions with high confidence in the zebrafish genetic model species for teleosts, aiming to uncover gene networks with notable enrichment in the list of candidate genes. Our identified network was found unlikely to be reproduced by randomly drawn gene selections, suggesting that biological relatedness can be elucidated from lists of candidate genes using the approach outlined in this study. An intriguing outcome is that the prominent feature in the identified network was epigenetic regulation in the form of chromatin remodeling, a biological process highly suspected to be involved in rapid domestication changes in fish (Konstantinidis et al. 2020; Piferrer, Miska, and Anastasiadi 2024). Additionally, we observed that some CpG islands, regions often targeted by methylation, were also implicated, highlighting the potential role of epigenetic modifications in the domestication process, a role supported by existing literature (Koch, Nuetzel, and Narum 2023). These findings further underscore the usefulness of the proposed method in moving beyond large‐effect genes in genome scan analyses, allowing for in‐depth examination of candidate gene lists and the architecture of polygenic traits.

Our results provide evidence regarding domestication in European seabass and were based on biallelic SNPs that demonstrated good differentiation in the PCA between farmed and natural‐origin populations (Figure 2). Previous studies using the same sequencing data have reached similar conclusions (Peñaloza et al. 2021; Villanueva et al. 2022), thereby highlighting the high genetic homogeneity among the studied natural‐origin populations and the genetic differences between the farmed populations. Consequently, the apparent varying degree of differentiation among farmed populations in the PCA (Figure 2) are likely due to domestication processes such as selective breeding and genetic drift, and may be associated with the number of generations each population has experienced under these pressures (Žužul et al. 2019). Nevertheless, the inclusion of farmed populations at possibly different stages of domestication in our dataset allowed us to draw universal conclusions about the genetic impacts of domestication, underscoring the importance of capturing both early and later generations. Consistent with the polygenic hypothesis of European seabass domestication, we detected 17 peaks spanning 11 linkage groups (LGs) with statistically significant differences in allele frequencies between farmed and natural‐origin populations, supported by two different genome scan methods (Figure 3). Several of the differentiated LGs have also been identified in previous studies on European seabass domestication using different datasets. These studies have highlighted LGs, including LG4, LG6, and LG14, with traits impacting growth performance, morphometric traits, stress tolerance and disease resistance in European seabass (Chatziplis et al. 2020; Louro et al. 2016; Massault et al. 2010). It thus appears that the detected LGs represent consistent, or at least frequent, targets of domestication in European seabass. However, we do not have details about the specific regions that were previously identified. In this context, our study also contributes to a gene‐level resolution aspect of this understanding.

To ensure the robustness of our conclusions, we employed genome scan analyses using two methods: one more liberal (Popoolation2) and one more conservative (BayPass) (Günther and Coop 2013). By selecting peaks identified by both methods, we improved our ability to infer true positive genomic regions potentially affected by domestication (Dalongeville et al. 2018; François et al. 2016). It is noteworthy that there were some inconsistencies between the two genome scan methods. *F*_ST_‐based and XtX‐based genome scan methods utilize distinct statistical models and assumptions, and they exhibit varying sensitivities to population structure, with XtX generally recognized for its lower false‐positive rate due to its ability to account for demographic history and population structure (De Mita et al. 2013; Günther and Coop 2013; Lotterhos and Whitlock 2014). The most noticeable difference between the two methods was identified at the peak of LG12 (Figure 3). By observing the estimated allele frequencies, we noted significant variation in allele frequencies within one farmed population, specifically fGRE_10, compared to others in this region. Additionally, within a 3 kbp window surrounding this peak, we observed 57 SNPs with up to 10 times higher read depth across all populations compared to the rest of the SNPs on LG12. According to the literature, such discrepancies in read depth could be attributed to segmental duplication (Numanagic et al. 2018). The choice to apply two methods—Popoolation2 and BayPass—proved effective in addressing depth variation in a manner consistent with the objectives of present study. An alternative option to address the increased coverage would be to first implement a maximum depth filter, as done by Spies et al. (2022), in their Pool‐Seq data of Pacific cod (Spies et al. 2022). However, in our study, we did not consider this necessary, as we relied on the consensus between two different methods that effectively mitigate this shortcoming. Therefore, it appears that in certain cases, additional factors could contribute to explaining the observed differences between the two genome scan methods.

With a robust dataset and a conservative methodology, we detected peaks influenced by domestication and highlighted crucial genomic regions, molecular processes, and pathways central to the evolutionary process of domestication. Notably, our findings may support the role of epigenetic mechanisms and chromatin remodeling in the domestication of European seabass. Previous studies have shown that epigenetic mechanisms are influenced by genetic background (Achilla et al. 2024; Lallias et al. 2021), with genetic variants in genes related to epigenetic processes also capable of affecting these same mechanisms (Cebrian et al. 2006; Maric et al. 2019; Potter et al. 2013). The chromatin‐modifying enzymes and entire chromatin organization pathways identified through our network analysis have been associated with fish domestication, observable as early as the first generation of adaptation to captivity (Le Luyer et al. 2017; Liu, Zhou, and Gao 2022; Milla et al. 2021; Whiteley et al. 2011). Chromatin remodeling plays a critical role in regulating gene expression by altering the accessibility of transcriptional machinery to specific genomic regions. This rapid and dynamic modulation of chromatin structure could facilitate swift phenotypic adaptations to domestication pressures, such as enhanced growth, improved stress response, and increased disease resistance (Best et al. 2018; Fellous and Shama 2019; Horsfield 2019; Labbé, Robles, and Herraez 2017; Varriale 2014). Notable genes with central roles in the identified network include those involved in heterochromatin assembly (*h2ax1*) and nucleosome formation (*hist2h2l*), as well as histone binding activity (*smarcc1a*) (Figure 6). The gene *h2ax1* is crucial for heterochromatin assembly, aiding in genomic stability by promoting the tight packing of DNA, which helps protect DNA from damage (Fernandez‐Capetillo et al. 2004). Furthermore, *hist2h2l*, which is orthologous to several human genes including *h2bc21*, plays a crucial role in nucleosome formation, which is vital for maintaining chromatin integrity. This gene encodes a variant of histone H2 that, together with other histone proteins, wraps around DNA to form nucleosomes (Talbert and Henikoff 2010). These structural units control DNA accessibility, crucial for fundamental cellular processes such as transcription, replication, and repair (Kouzarides 2007). Additionally, *smarcc1a*, a component of the SWI/SNF chromatin remodeling complex, binds to histones, allowing for the repositioning or restructuring of nucleosomes, thus modulating the chromatin landscape to either repress or activate gene expression (Auman et al. 2024; Bieluszewski et al. 2023). Together, these genes coordinate the dynamic organization of chromatin, ensuring precise control over gene expression and maintenance of genomic integrity. These observations suggest that chromatin remodeling and epigenetic mechanisms may play a pivotal role in the contemporary adaptation of European seabass to domestication pressures, potentially by facilitating swift phenotypic changes through the dynamic regulation of gene expression and maintenance of genomic stability (Koch, Nuetzel, and Narum 2023). However, we did not provide direct evidence of how these changes translate into functional gene expression modifications. Future studies employing transcriptomic and epigenomic approaches are necessary to elucidate the specific pathways through which these epigenetic mechanisms influence phenotypic traits, thereby validating their role in the domestication process.

Moreover, the identification of nine CpG islands as neighboring features of the peaks within our recognized linkage groups supports the idea that epigenetic mechanisms play a role in the domestication process of European seabass. Recent studies suggest that phenotypic changes can occur very early in the domestication process—often within the first generation in captivity—indicating that epigenetic mechanisms may significantly influence the early onset of domestic traits (Podgorniak et al. 2022; Koch, Nuetzel, and Narum 2023). In this context, CpG island methylation patterns have been associated with the domestication process in fish species, particularly in their role in regulating growth‐determining genes, such as those involved in muscle growth, immunity, and dietary responses (Moore, Le, and Fan 2013; Moulistanos et al. 2023; Podgorniak et al. 2022; Koch, Nuetzel, and Narum 2023). It has even been suggested that these epigenetic modifications may precede artificial selection and facilitate fish adaptation to farming conditions (Podgorniak et al. 2022). Overall, the identification of CpG islands and their association with early domestication traits, combined with the potential role of chromatin remodeling in regulating gene expression, underscores the critical influence of epigenetic mechanisms in shaping the rapid adaptation of European seabass to domestication pressures that needs to be investigated in more detail. Specifically, combining genomic, epigenetic (such as DNA methylation and chromatin profiles), and gene expression data would offer a more direct evaluation of the adaptive significance of the epigenetic effects in the domestication process.

The Gene Ontology enrichment analysis indicated that “oxidoreductase activity” is a molecular function potentially influenced by domestication (Table 2). A comparable observation was made in zebrafish research, examining the impact of domestication selection on lab strains versus natural‐origin populations (Whiteley et al. 2011). The literature indicates that oxidoreductase activity plays a significant role in various domestication‐related processes, including metabolism, detoxification, and adaptation to environmental stress in different fish species (Ao et al. 2015; Kolesnikova et al. 2022; Windisch et al. 2014). Given these critical roles of oxidoreductase activity, it can be hypothesized that these enzymatic processes may facilitate the domestication of fish species by promoting rapid physiological changes, including more efficient energy utilization and improved stress responses. For instance, among the identified candidate genes, the cytochrome P450 2 (CYP2) gene family and *hsd17b* genes are implicated in metabolism and synthesis of steroid hormones (Liu et al. 2024; Uno, Ishizuka, and Itakura 2012), *mmachc* is involved in vitamin B12 metabolism (Sloan et al. 2020), while *aldh8a1* with a highly conserved sequence on vertebrates is linked with diverse functions related to aldehyde metabolism (Holmes 2017). *Pyroxd1*, and *txnrd2.2* are associated with responding to oxidative stress (Espino et al. 2022; Li et al. 2022). Intiguingly, *tet3*, along with *alkbh3*, are also associated with in epigenetic regulation (Bian et al. 2019; Gonzalez et al. 2023; Wu and Zhang 2017; Yuting, Quan, and Liang 2018). The traditional enrichment analysis also revealed that some genes, which were enriched for redox reactions, were associated with epigenetic mechanisms.

In summary, our study substantially enhances understanding of the genetic architecture of domestication in European seabass. By analyzing populations with diverse genetic backgrounds and varying stages of domestication, we provided a detailed assessment of the genetic impacts and genomic architecture underlying domestication. Our findings pinpointed critical genomic regions and elucidated the molecular processes and pathways central to this evolutionary process. Importantly, our results support a polygenic model of domestication in European seabass, highlighting the significant roles played by epigenetic mechanisms, both chromatin remodeling and methylation activity, in enabling contemporary adaptation. These insights not only enhance our understanding and advance our comprehension of how selective pressures affect genetic diversity but also illustrate a potential complex interplay between genetic and epigenetic factors in shaping phenotypic traits of commercially important fish species. Future research could further elucidate the role of epigenetic regulation in domestication and provide deeper insight into how genomic and epigenetic changes contribute to rapid adaptation in response to domestication pressures.

## Author Contributions

**Aristotelis Moulistanos:** data curation (equal), investigation (equal), methodology (equal), project administration (supporting), visualization (supporting), writing – original draft (equal), writing – review and editing (equal). **Konstantinos Papasakellariou:** methodology (supporting), writing – review and editing (supporting). **Ioannis Kavakiotis:** methodology (supporting), writing – review and editing (supporting). **Konstantinos Gkagkavouzis:** methodology (supporting), project administration (equal), visualization (supporting), writing – review and editing (supporting). **Nikoleta Karaiskou:** methodology (supporting), project administration (supporting), writing – review and editing (supporting). **Efthimia Antonopoulou:** investigation (supporting), methodology (supporting), supervision (supporting), writing – review and editing (supporting). **Alexandros Triantafyllidis:** conceptualization (supporting), funding acquisition (supporting), investigation (supporting), methodology (supporting), project administration (supporting), supervision (equal), writing – review and editing (supporting). **Spiros Papakostas:** conceptualization (lead), data curation (lead), formal analysis (lead), funding acquisition (lead), investigation (equal), methodology (equal), project administration (equal), resources (lead), software (lead), supervision (equal), validation (lead), visualization (equal), writing – original draft (equal), writing – review and editing (lead).

## Conflicts of Interest

The authors declare no conflicts of interest.

## Supporting information


Table S1

## Data Availability

Raw sequence reads are available in NCBI's Sequence Read Archive (SRA) under accession number PRJEB40423. All the scripts developed for the data analysis are available on GitHub: https://github.com/spirospapakostas/PoolSeq_Dlabrax_domestication.
