# Supplementary material for: Genomic Signatures of Domestication in European Seabass (Dicentrarchus labrax L.) Reveal a Potential Role for Epigenetic Regulation in Adaptation to Captivity
Source: Ecol Evol. 2024 Dec 3;14(12):e70512. doi: 10.1002/ece3.70512 (PMC11612516; doi:10.1002/ece3.70512)
Supplement: Supplementary file 1 — Table S1 [file ECE3-14-e70512-s001.docx]

TABLE S1. Information about the position of ‘suggestive’ and ‘highly suggestive’ SNPs, differentiation values (Fst and XTX) with adjusted p-values and annotated genes and regulatory elements detected in European seabass for regions within 100 kbp of the peaks with the maximum differentiation in each case.

| **Linkage group** | **pos** | **Suggestive (S)**  **or**  **Highly suggestive (HS)** | **Popoolation2** | | **Baypass** | | **Genes and regulatory regions within a 100 kbp window around each SNP**  **(* genes in which the identified SNP located)** |
| --- | --- | --- | --- | --- | --- | --- | --- |
|  |  |  | **Fst** | **log(1/p-val)** | **XtX** | **log(1/p-val)** |  |
| LG4 / HG916844.1 | 19571521 | HS | 0.322 | 6.853 | 104.345 | 7.936 | *hsd17b7; serbp1a; il12rb2; LOC110438375; LOC103909477; cyp2p10; cyp2p7; cyp2n13; cyp2p6; hook1*; si:dkey-183n20.15; pfas; ipo13b; muc5.1; LOC562098; mmachc* |
| LG6 / HG916846.1 | 6425860 | S | 0.223 | 4.851 | 100.871 | 7.300 | CpG island*; lrrc4ca* |
|  | 6502417 | S | 0.182 | 4.472 | 98.752 | 7.222 |  |
|  | 18949747 | S | 0.233 | 5.643 | 71.043 | 3.456 | *abhd2b; rlbp1b; isg20; pdia3; ckmt1; ticrr; si:ch1073-281m9.1*; alkbh3; hsd17b12a* |
| LG8 / HG916848.1 | 22485140 | S | 0.148 | 3.637 | 90.755 | 5.538 | *LOC559196; CBX1; nfe2l1b; D5F01_LYC18948; copz2; prr15la; LOC110437935* |
| LG9 / HG916849.1 | 9102523 | HS | 0.249 | 5.154 | 94.370 | 6.232 | *hipk3b* |
| LG10 / HG916827.1 | 7906972 | S | 0.262 | 6.23 | 70.53 | 3.029 | *imp3; slc35a3a; fam78ba; Cmpk; bcl6ab; zmym4.2; cldn1; cpn2; fkbp1b; her6; atp13a3*; ncl; gk5; atp1b3a; grk7a; fbxo36b; agfg1b; si:dkeyp-13a3.3; mffa; stk25b* |
| LG14 /  HG916831.1 | 4359799 | S | 0.162 | 3.671 | 92.250 | 5.725 | *vps11; hyou1; si:ch73-261i21.5; hist2h2l;* CpG island*; h2ax1; hspa8b; LOC110438375; hspa8b; jhy*; bsx;* CpG island*; lim2.1* |
| LG16/  HG916833.1 | 1228405 | S | 0.208 | 6.071 | 81.415 | 3.957 | CpG island*; smarcc1a;* CpG island*; si:ch211-215k15.4*; dpy19l1l* |
| LG17 / HG916834.1 | 9858550 | S | 0.23 | 5.35 | 73.542 | 3.116 | *map7b; hbs1l*; armc1l; mtfr2; slc39a9; pde7a; myb; aldh8a1* |
| LG20 / HG916840.1 | 22630001 | S | 0.138 | 3.203 | 85.279 | 5.056 | CpG island*; txnrd2.2*; gnb1l; tbx1* |
|  | 23126527 | S | 0.148 | 3.748 | 88.323 | 5.408 | *si:ch211-244o22.2*; CpG island*; kat6a*; ap3m2; tet3* |
|  | 24100922 | S | 0.160 | 4.133 | 90.129 | 5.422 | *bicdl1; rab35a; gcn1; vamp5; vamp8; ncaph*;* two CpG islands |
| LG24/ HG916842.1 | 3483292 | HS | 0.236 | 6.221 | 106.208 | 8.529 | *creb1b; mettl21a; ccnyl1* |
| LGx / HG916850.1 | 6263532 | S | 0.226 | 5.402 | 72.984 | 3.537 | *etnk1; pyroxd1; Iapp; slco1e1; slco1d1* |
|  | 6268715 | HS | 0.239 | 5.583 | 90.766 | 5.969 |  |
|  | *8424464* | *HS* | *0.273* | *6.405* | *99.806* | *7.269* | itgbl1* |
